# SYMPTOMS CONTRIBUTING TO SLEEP PROBLEMS IN OLDER ADULTS WITH TYPE 2 DIABETES

**DOI:** 10.1093/geroni/igac059.1775

**Published:** 2022-12-20

**Authors:** Min Jung Kim, Cynthia Fritschi, Eunhee Cho

**Affiliations:** College of Nursing, Yonsei University, Seoul, Seoul-t'ukpyolsi, Republic of Korea; University of Illinois Chicago, Chicago, Illinois, United States; College of Nursing, Yonsei University, Seoul, Seoul-t'ukpyolsi, Republic of Korea

## Abstract

Sleep problems are common in older adults. Those with diabetes are more vulnerable to sleep disorders since diabetes-specific symptoms can interfere with sleep quality. Yet little is known which diabetes symptoms most strongly affect sleep in older adults. This study aimed to examine the associations between diabetes symptoms and sleep and to identity the symptoms that most strongly disrupt sleep in older adults in the United States. Diabetes symptoms were assessed using the Diabetes Symptom Checklist-Revised. Sleep impairment and sleep disturbance were self-reported using The Patient-Reported Outcomes Measurement Information System. Demographic (age, sex, race/ethnicity) and other variables (body mass index, depressive symptoms, diabetes duration, glycemic control) were also assessed. Multivariate regression analyses were used with standardized coefficients. A total of 82 adults aged ≥ 60 years were included (mean age = 68.32 ± 5.29 years, White 76.83%, female 56.1%). After controlling for demographic and other variables, increased hypoglycemia (β = .35), hyperglycemia (β = .38), fatigue (β = .65), cognitive (β = .48), and ophthalmologic (β = .25) symptoms and neurological pain (β = .42) significantly increased sleep impairment. Of these, fatigue was the strongest contributor to sleep impairment. Similarly, increased hyperglycemia symptoms (β = .30), fatigue (β = .34), and neurological pain (β = .37) significantly increased sleep disturbance while neurological pain was the strongest contributor. To improve sleep quality of older adults with diabetes, their diabetes symptoms should be comprehensively assessed, and potential contributor to poor sleep such as increased fatigue and neurological pain should be addressed.

